# Unravelling the dynamics of child sexual exploitation material circulation on the Dark Web

**DOI:** 10.1371/journal.pone.0306516

**Published:** 2024-07-24

**Authors:** Pramod Divakarmurthy, Bruno Requião da Cunha, Jean Fernando Passold, Marcos Oliveira, Ronaldo Menezes

**Affiliations:** 1 BioComplex Laboratory, University of Exeter, United Kingdom; 2 Global Investigations, TRM Labs, San Francisco, California, United States of America; 3 Santa Catarina Superintendency, Federal Police, Florianópolis, Brazil; 4 Department of Computer Science, University of Exeter, United Kingdom; 5 Department of Computer Science, Federal University of Ceará, Fortaleza, Brazil; Bar Ilan University, ISRAEL

## Abstract

In recent years, there has been a significant increase in the detection of child sexual exploitation material (CSEM), with perpetrators increasingly turning to advanced encryption technologies to conceal their activities. This study delves into data from a Brazilian Federal Police operation on the Tor network, aimed at disrupting these illicit activities. We uncovered patterns indicating strong user preferences for certain content categories, suggesting the existence of distinct groups with shared interests. Additionally, our findings reveal consistent activity patterns among users, including specific 24-hour, 12-hour, and 6-hour consumption cycles. This research offers insights into the online behavior related to CSEM, providing a foundation for further investigation and the development of effective policy measures.

## Introduction

In the past decade, exposure of child sexual exploitation material (CSEM) has reached concerning levels, with the volume of detected content rapidly increasing year by year [1]. In fact, child pornography is a multi-billion dollar abhorrent industry rooted in child abuse [2]. In most countries, this act is recognised as a grievous crime, yet law enforcement agencies often encounter difficulties in identifying and apprehending those involved in underground networks that exploit children. These challenges are compounded by variations in legal definitions of child exploitation material across different jurisdictions. Individuals engaging in illegal activities often participate in concealed networks, connecting people who abuse children with those seeking to access illicit media. These networks operate similarly to markets, where the demand for such materials drives their production and distribution [3]. With today’s level of Internet accessibility and easy access to social media platforms, the trading of content occurs mostly on-line, causing child exploitation material to quickly proliferate [4–6].

In the past decade, offences related to child sexual exploitation material have seen a staggering 233% surge, primarily driven by the growth of the Internet [7]. The Internet provides both efficient and seemingly anonymous access to such content and an environment where online forums facilitate interaction among individuals with shared interests, further legitimising their sexual interests and activities [5, 8]. Individuals involved in illegal activities frequently use the Dark Web—a hidden part of the Internet accessible only through specific software—to conceal their digital trails and identities, with an alarming 80% of the Dark Web’s activity linked to child exploitation material [9]. This secrecy complicates efforts by researchers and law enforcement to understand how individuals engage in illicit activities online.

Our understanding of Dark Web offending behaviours lags significantly behind our grasp of other criminal patterns. For many types of crimes, we have discerned temporal and spatial regularities, identified specific circadian rhythms [10], mapped spatial concentrations [11], and analysed dynamics within criminal networks [12] and organised crime [13]. These insights have empowered law enforcement with more robust investigatory tools and have informed more effective intervention strategies. However, this level of understanding starkly contrasts with our current grasp of CSEM consumer behaviours.

While some progress has been made online, such as identifying a concerning trend of increasing severity in the sexual acts depicted in consumed material [14], and profiling users’ communication patterns on large Dark Web forums to assist law enforcement [15], these developments only skim the surface. A comprehensive, granular understanding of individual offender dynamics within this realm still remains a significant challenge.

Of course, it is not to say that insights into the behaviours and backgrounds of individuals involved in CSEM offences are lacking. Gottfried et al. [16] underscore the demographic characteristics and behaviours observed among individuals engaging in online sexual offences, highlighting a notable presence of white males with fewer prior felony arrests than those found in contact offence cases. This study highlights distinct risk factors, such as an interest in children combined with antisocial attitudes, and addresses the challenges in assessing recidivism due to the absence of specifically tailored assessment tools. Faust et al. [17] provide a study between individuals possessing child pornography and those committing contact offences against children, revealing significant differences in demographic backgrounds and criminal histories. Individuals with offences related to child pornography showed fewer instances of previous criminal activities and higher levels of employment and education prior to incarceration. Notably, they demonstrated substantially lower recidivism rates, suggesting they have different needs for rehabilitation and risk profiles. Babchishin et al. [18] observed that individuals committing online sexual offences often are slightly younger and more likely to be Caucasian, with a higher capacity for victim empathy compared to their offline counterparts. Additionally, they exhibit a higher degree of sexual deviancy and a lower tendency towards impression management. Both groups, compared to the general population, reported higher incidences of childhood abuse. The findings suggest that individuals involved in online offences may possess greater psychological barriers and self-control, which potentially prevent them from acting on their deviant interests, in contrast to individuals involved in offline offences.

In this paper, looks at general patterns from a data-science and network science point of view. We build a child-pornography space of interactions in the dark Web to examine the consumption and production dynamics of child pornography. For such purpose, we use a combination of temporal analysis, content analysis using unsupervised machine learning techniques (i.e., clustering), and network analysis. We rely on a dataset from the *Dark Net Operation* [19, 20], an inquiry of the Brazilian Federal Police which tracked a child-pornography forum in the Tor network. With two phases, the investigation lasted from 2014 to 2016 and resulted in hundreds of search warrants and in almost 70 arrests, with 6 children rescued from abusive situations. On the on-line forum, the users exchanged child abuse media and sexual abuse experiences. The data consists of user’s activity on the forum such as posts, comments, and viewing. Also, each post in the forum was classified into categories based on criteria developed by the users themselves [21]. Notably, for these individuals, categorisation of their collection is known to be an obsession [22]. Therefore, the data enable us to explore how categories emerge from the social context of these individuals’ activities.

## Materials and methods

### Data collection and sources

The data from the online forum acquired by the Brazilian Federal Police was structured inside 67 categories, containing a total of 9,367 posts and 6,248,719 views made by 9,280 users. Inside the posts, users express their opinion and share CSEM content. The CSEM were often shared as an external link (URLs) to encrypted files containing photos and videos, but sometimes users upload CSEM directly inside their posts. The direct upload of files inside posts summarises 789 CSEM files. Every post, is associated with an identifier, user, category and the date/time when it was posted. Similarly, every view can be mapped to a user, post and the date/time when the post was viewed (We have made the anonymised dataset available [21])

The Combating Paedophile Information Networks in Europe (COPINE) classification system comprises 10 levels, designed to categorise images related to adult sexual interest in children [4]. Levels 1–6, includes common pictures of children, which, when viewed or stored inappropriately, can fall within these categories. Blurred boundaries between these categories can occur, with overt sexual intent being the critical factor. Levels 7 and above involve pictures depicting sexual assault or rape in progress and are universally considered child pornography, irrespective of the offender being visible in the scene or not. This gradation (levels) allows for a more precise understanding and assessment of the content, facilitating its use in both academic research and legal proceedings.

The significance of the COPINE scale extends beyond its academic origins, as it has become a vital tool for law enforcement agencies worldwide in the fight against child exploitation (like the Brazilian federal agents have done in this case). By providing a common language and standard for evaluating the severity of abuse depicted in images, the scale aids in the investigation, prosecution, and sentencing of individuals involved in the creation, distribution, and possession of child sexual abuse material. Here, the COPINE classification is applied on 67 categories by federal agents; they have assigned a COPINE scale to each of the categories based on the content of the categories.

The original data and images were handled only by Brazilian Federal Police Agents (including the author JFP, who was the Operation Darknet lead investigator) with legal clearance to do so, and under the supervision of a Federal Judge and Prosecutors. All data handling was in accordance with the Brazilian law for data protection (Act No. 13,709 from 2,018), the Brazilian individual rights act (Brazilian Constitution from 1,988), the Brazilian Criminal Code (Act No. 2,848 from 1,940), the Brazilian Criminal Procedure Code (Act No. 3,689 from 1,941), and the Brazilian Federal Police internal procedures. All data was analysed by the authors only after a complete cryptographic anonymisation procedure, making it impossible to identify the individuals involved. This means that the authors working in these analyses did not have any access to the raw materials, including images. The anonymised data is available upon request via the authors.

### Analysis

#### Descriptive analysis

To build the child-pornography space of interactions in the dark Web, we first filtered out less active users because many of the analysis here require data for us to have a trend. We analyse the growth rate of user’s activity on the forum for a given period of time. Using frequency distribution, we try to understand how the users behave whilst in the forum. We can specifically study the distribution of the number of images viewed by users and the number of images posted by users. Based on user’s activity on the types of image contents viewed and posted, we can understand whether the users are aligned to any specific categories of interest.

#### Temporal analysis

**Fig 1:**
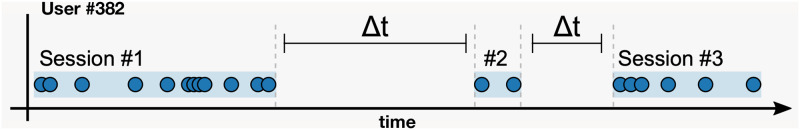
User #382’s activity on the forum for a given time frame. Shows the period of time the User #382 is active on the form. If a user is inactive for one hour or more, Δt > 1h, any future activity is attributed as a new session. In this figure, we have an example with 3 sessions being displayed.

A session is a list of user interactions (i.e., views, posts) on the forum that take place within a give time frame. For example, Fig 1 shows a single user’s activity with multiple sessions and multiple events during a single session. We consider an event, activities such as viewing an image or posting an image or any kind of social interactions over messages. If the user is inactive for an hour or more, any future activity is attributed to a new session; we choose this threshold based on the inter-event time distribution. The frequency decreases at 1h; thus we justify this value as the duration interval that separates sessions. Observing user’s activity by sessions help us understand users’ behaviour and the patterns based on the time spent on the forum.

#### Content analysis

Since images are classified into several categories based on their contents, we can examine whether users are aligned with the content from specific categories. Given such a content classification, we can generate feature vectors for each user, which can be used to cluster users into several groups using a k-means algorithm. This will allow us to analyse the behaviour of users within each group and discern the differences between the groups.

#### Network analysis

Users display a social behaviour on the forum, by sharing information and media in the form of posted messages. These discussions among users allow them to explore images from other categories. As users navigate between categories, the relationship between categories starts to appear. We applied network analysis to understand the relationship between these categories. Two categories are linked if the content in them are consumed by the same user, regardless of when they were consumed. With this network, we analysed the dynamics of the users in the forum and how they navigate in this space. To further study the strength between two categories, we calculate the relative risk (RR). Relative risk is often used to compare the risk, likelihood or a chance of an event occurring between two groups (exposed group and non-exposed group). The RR is calculated by

$$\begin{array}{c} RR_{ij}=\frac{C_{ij}N}{P_{i}P_{j}}, \end{array}$$

where *C*_*ij*_ is the number of users who consumed from both the categories, *N* is the total number of users in the population and *P*_*i*_ and *P*_*j*_ are the prevalence of categories *i* and *j*.

The interpretation of the values of *RR*_*ij*_, is that values greater than 1 indicates a statistically significant likelihood of users engaging with content across both categories *i* and *j*, revealing a strong associative bond. Conversely, an *RR*_*ij*_ equal to 1 suggests a lack of association, indicating that interactions with these categories occur independently of each other. Should *RR*_*ij*_ fall below 1, this denotes a likelihood of cross-category engagement lower than expected, highlighting a weaker linkage. This analysis is important for elucidating user content navigation and preference, as well as interaction between categories, offering insights into the dynamics of forum engagement in the context of our study.

## Results

**Fig 2:**
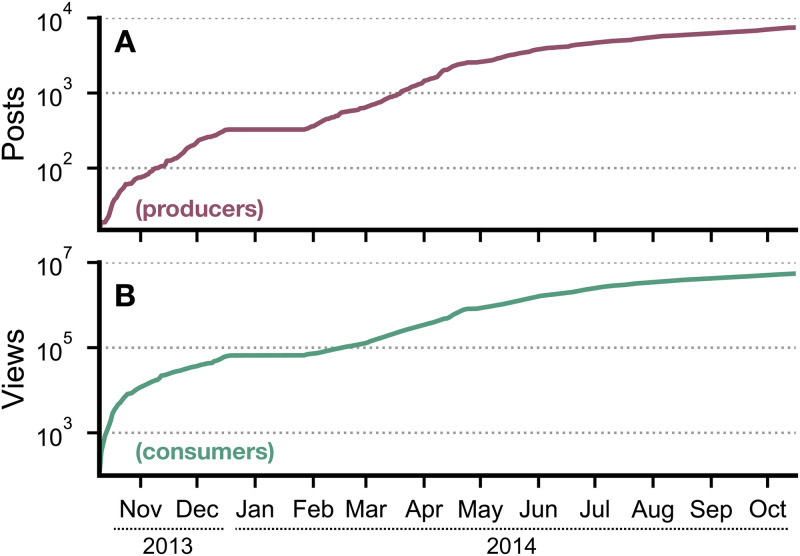
Cumulative count of posts and views on the forum. User’s activity over a year, starting from November 2013 until October 2014. **(A)** Cumulative growth rate of posts. **(B)** Cumulative growth rate of views. It is worth noticing that no user is filtered out here (and in no analyses until Fig 5).

We examined the consumers’ and producers’ activity growth rate from November 2013 to November 2014 as shown in Fig 2. The cumulative count shows an initial fast increase in number of views and posts in the early stages, followed by a substantial growth from Feb-14 to May-14. The increase in the number of posts was followed by an increase in the number of views.

**Fig 3:**
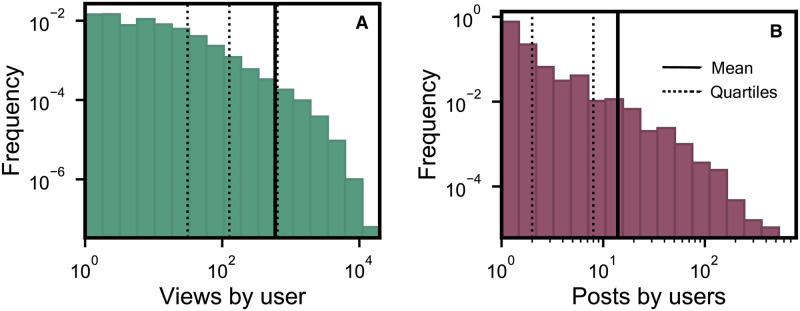
Posts and Views by users. User’s activity in the forum varies based on their interest in viewing or posting contents. **(A)** shows the frequency of users to the number of contents viewed. **(B)** shows the frequency of users to the number of contents posted.

User’s activity in the forum varies based on their interest in viewing or posting contents. Fig 3(A), shows the frequency of users on the forum to the number of contents viewed. The data shows that a subset of forum users visit the forum infrequently, resulting in low activity levels, with view counts below 10. The majority of users have view counts ranging from 10 to 1000, with around 400 users on average having roughly 700 views. Conversely, there is a group of users who exhibit consistent forum engagement and maintain high levels of activity during their visits, with view counts ranging from 1K to 10K. Similarly, we also analysed the user’s activity on the forum based on the contents posted. Fig 3(B), shows the probability distribution of posts by users. A large portion of producers tend to submit posts fewer than 5, but on average, users post approximately 10 contents per user. However, there are very few users who actively post contents on the forum; the distribution of posts is long tail. These individuals are participants in an online forum that is under investigation for illegal activities. They are considered key figures of interest by law enforcement and intelligence agencies due to their roles in facilitating the exchange of illegal images and encouraging participation in these exchanges among other members of the forum.

**Fig 4:**
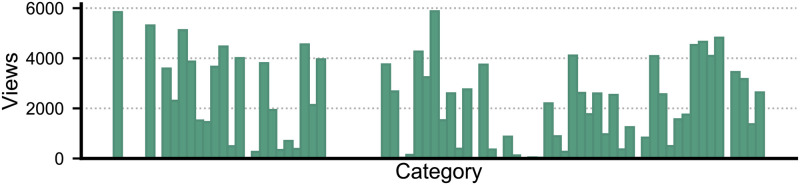
Views based on categories. Shows the users’ activity of viewing contents in selected categories. Views are concentrated on a few categories, while others have very few views.

We identified 67 different categories for contents, which were classified by the users themselves. Clearly, we can see from the Fig 4, that the majority of the users have viewed contents published from a selected number of categories. The images in these categories are driving the traffic to the forum and attracting new users. While, there are a number of categories in which the contents are viewed fewer than a few hundred times.

**Fig 5:**
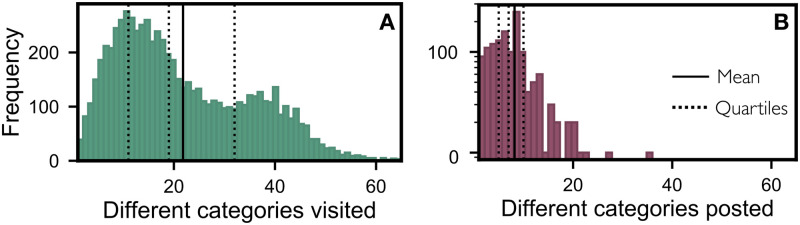
Activity based on unique categories. Analysis of user’s activity on number of unique categories visited. **(A)** shows the frequency of users to count of unique categories visited to view contents (consumer). **(B)** shows the frequency of users to count of unique categories visited to post contents (producer).

The activity of a user on the forum can also be analysed by considering the number of distinct categories they have visited. As users spend more time on the forum and explore their interests, they navigate from one category to another, providing valuable insight into their level of engagement. Fig 5 shows the frequency of users based on the number of different categories visited to view contents. It also indicates that the majority of users stay within a certain number of categories. On average, users visit approximately 20 different categories over a period of time. Only a few users explore many or all the categories. Similar behaviour was observed on user activity of contents posted in different categories. Fig 5, shows many users prefer to post contents only in certain categories. On average, users posted in approximately 10 different categories.

### Temporal analysis

**Fig 6:**
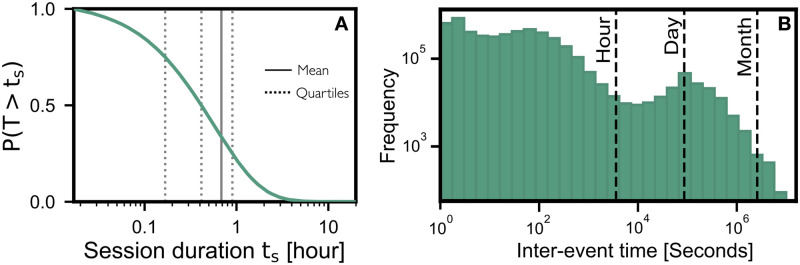
User’s activity on the forum for a given time frame. This shows the period of time the user is active on the forum. If a user is inactive for 60 minutes or more, any future activity is attributed as a new session. **(A)** illustrates the likelihood of the amount of time spent during a session; the average session is around 40 minutes in duration (compared to around 10 for a mainstream porn site [23]. **(B)** represents the frequency of users regarding the time elapsed between two events.

The examination of users’ activities within a specified time frame aids our comprehension of their conduct and their forum usage patterns. We classified the users’ activities into sessions, considering events to be separate sessions if there was no activity for a period exceeding 1 hour. The amount of time spent during a session on the forum varies from user to user. Fig 6(A) shows the probability of session duration. We observed that the probability of a user spending at least 10 minutes on the forum was close to 85% and the probability of spending almost 1 hour was almost 25%. We also noticed that there are sessions which go over 2 hours, but occurrences of those are very rare. The mean duration of a session was observed to be roughly 40 minutes, which surpasses the typical session length of about 10 minutes on a mainstream pornographic website [23].

To further understand the users’ activities on the forum, we analysed the time between each event (inter-event time). The inter-event time helps us understand users’ engagement in their activities in the forum. Fig 6(B), shows the frequency distribution of time between two events. Most of the users spend anywhere between 1 minute to 1 hour between two events. We also observed, that there are a high number of users who resumed their activity the very next day. There are also few users who have inter-event time of over 1 month between each event.

**Fig 7:**
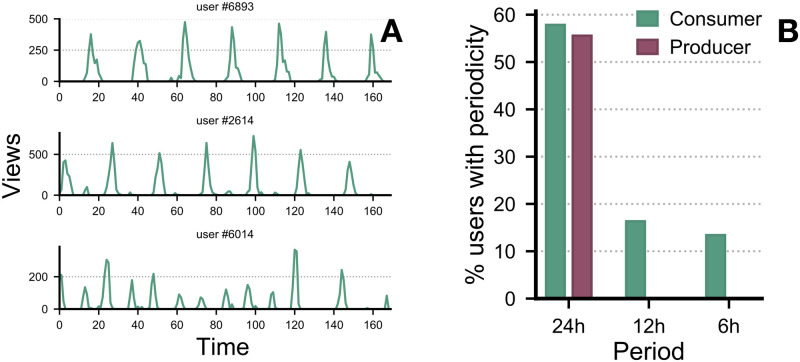
Temporal regularity of users aggregated over a week. **(A)** shows the temporal regularities of some individual users. **(B)** shows the percentage of users to the periodicity pattern.

By analysing the temporal regularity of individual users, we can observe their activity on the forum for a given period of time, aggregated over a week. Fig 7, shows the temporal regularities of individual users. We see an example in which the User #6893 is a frequent visitor and their activity pattern shows a periodicity of 24 hours. Similarly, Users’ #2614 and #6014 activity pattern shows a periodicity of 12 hours, with User #6014 having a strong visitation pattern o 12 hours. Fig 7(A) shows the temporal regularities of all users who view (consumer) and post (producer) contents. It should be noted that producers operate on a 24-hour periodicity, while consumers also have a 24-hour periodicity. However, some consumers have periodicities of 12 or 6 hours as well.

Our analysis, categorising user activities into sessions and examining inter-event times, delineates clear patterns of engagement. We observed periodicity in user activities, with distinct 6, 12, and 24-hour cycles emerging from the data. These temporal regularities, evident across both content consumers and producers, with producers predominantly adhering to a 24-hour cycle and consumers more periodicities including 6 and 12 hours, align with literature findings on human activity cycles [24–26]. Such periodic patterns in forum engagement not only reinforce the significance of ultradian and circadian rhythms in shaping human behaviour but also underscore the importance of considering these temporal dimensions in understanding user interaction with digital platforms.

### Content analysis

Transitioning from the exploration of temporal patterns in user activity, we now delve into the content preferences among users to uncover the diversity of their interests. This analysis employs clustering and entropy to quantify the variability in content consumption and production across different categories, offering insights into the degree of uniformity or heterogeneity in user preferences. By examining the categories frequented by producers and consumers, we aim to establish a deeper understanding of the dynamics at play within the forum’s ecosystem.

**Fig 8:**
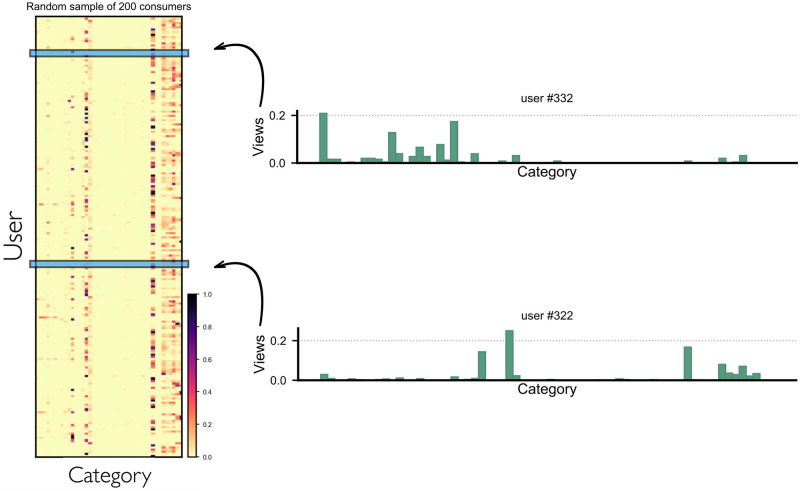
User-Category cluster analysis. The distribution of content views across various categories is displayed for a sample of 200 randomly selected users. Analysis reveals significant discrepancies in category preferences between User #332 and User #322.

We examined the categories visited by each user to understand if users share similar preferences. To illustrate, Fig 8 shows a sample of 200 users randomly selected and a detailed overview of two users showing the percentage of views in each of those categories. Note that the preferences between the two users vary significantly, and this can also be observed in the heatmap. Dark colour indicates higher number of views for contents from those categories, whereas, light red or yellow colour indicates lower number of views. Looking at the sample of users, we can see that contents from certain categories are viewed by most users, while other categories are viewed only by few users. However, this does not answer, if there is homogeneity among users.

**Fig 9:**
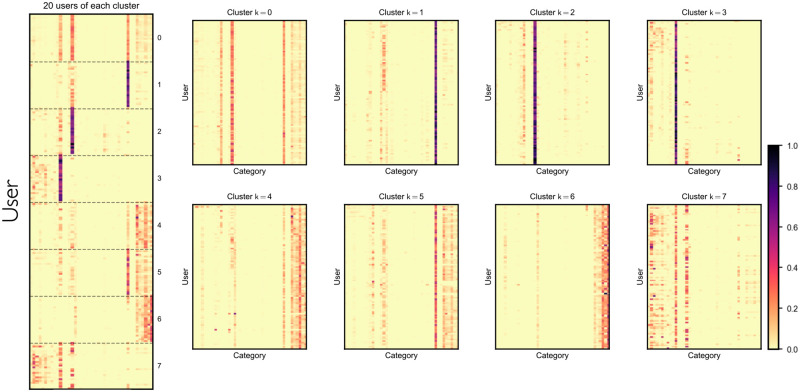
Analysis of users by clusters. Shows the users from each cluster and their content preferences.

**Fig 10:**
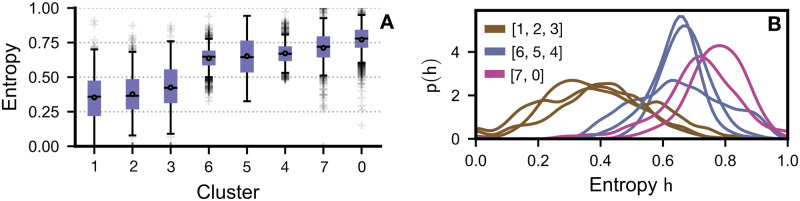
Entropy of clusters. Based on the entropy, three distinct groups can be discerned, ranging from broad preferences (high entropy) to narrow preferences (low entropy). The entropy distribution for each cluster is presented in both **(A)** and **(B)**. The shape of the distributions can be more easily observed in **(A)**.

We applied k-means to group users with similar content preferences together. After several experiments, we determined that the optimal number of clusters needed for our task was 8 clusters. Fig 9 shows how users are clustered into groups, with the colour shade (dark to light) indicating the number of views. Only for illustration purpose, we have chosen 20 users from each cluster. We see that in cluster 1, 2 and 3, contents from only a selected number of categories are viewed by users. Clearly, users in these clusters have a strong preference towards certain categories. Whereas, the content preferences of users seem to diversify in other clusters. These preferences are reflected in the entropy of the categories for each of the clusters shown in Fig 10 (see S1 Appendix for details on the entropy calculation).

### Network analysis

Building on our analysis of temporal patterns and content preferences, we next explore the structural connections within the content network to unveil how different categories interrelate, as highlighted in Fig 4. This progression allows us to map the landscape of content flow and user interaction, providing insights into the complex web of category relationships and their implications for user engagement and forum dynamics.

**Fig 11:**
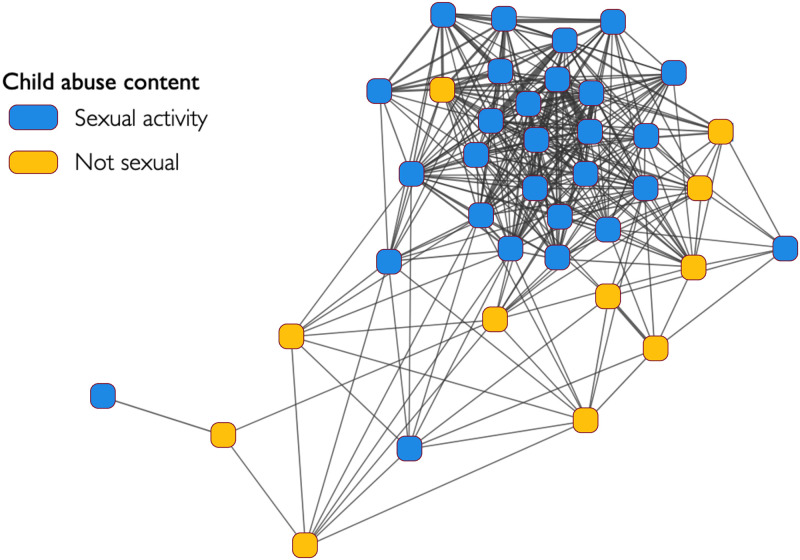
Network of categories. The present network comprises a collection of categories that are established based on the content of child abuse. It should be acknowledged that, out of the 67 recognised categories, some have been removed by filtering of edge values. This, in turn, has led to the removal of isolated nodes. The purpose here is just to demonstrate that sexual content categories tend to form the central part of the network, while non sexual content is peripheral.

After constructing the network (as described earlier), we calculated and applied the relative risk between two categories to quantify the distance between them. To study the core of the network, we filter all links with *RR* < 1. We applied the COPINE classification [4] on the categories to segment contents with “Non sexual” (low level of severity; COPINE grade up to 6) and “Sexual” (high level of severity; COPINE grade from 7 to 10) as defined in Table 1 in S1 Appendix. Fig 11, shows the network of categories where the nodes are the different categories and links showing common interest from users and the colour indicates the segment to which the categories belong. What this network reveals is that the core of the network is composed of sexual activity categories.

## Discussion

In the context of the significant rise in child sexual exploitation material (CSEM), descriptive analytics emerges as a pivotal tool within our study. This approach is not only foundational in identifying offender behaviours and patterns but is also essential due to the current scarcity of behavioural models in this domain. Our analysis, rooted in data from a Brazilian Federal Police operation, reveals crucial insights into the temporal patterns, content preferences, and the network of relationships among these content categories. These findings lay the groundwork for future research and have immediate implications for law enforcement strategies and policy formulation, offering a pathway toward more effective CSEM combat strategies.

We examined the material circulation (consuming and producing) growth rate and found it to increase substantial after gradual growth in the early stages. User’s activity on the forum generally varies based on their interest. There are users who visit occasionally and those who are very active on the forum. We also found similar trends among producers. With over 67 different categories, users clearly have preferred categories and tend to spend more time on contents from those categories. However, only a few number of categories drive traffic and also attract new users. The analysis of users’ activities where events are grouped into sessions helps us understand their behaviour and the pattern of how they spend time on the forum. We noticed that the average session time was approximately 40 minutes and that very few activities last more than 2 hours. Fig 5(A) highlights the sigmoidal characteristics of session lengths. The choice of 1 hour is then based on these lengths and also in line with the literature that also analysis human traces in hourly periods [27]. A wealth of research in this domain underscores that choosing hourly intervals strikes an optimal balance between the granularity and manageability of data, especially for analysing activities with notable daily fluctuations. It is commonly observed that time frames such as hourly, monthly, and weekly are preferred for the study of human activities [28].

Our observations further reveal a substantial proportion of users re-initiating their activity within a 24-hour cycle, with a predominant periodicity reflecting daily engagement patterns. Notably, distinct temporal cycles of 6 and 12 hours were predominantly identified among content consumers, diverging from the more uniform 24-hour periodicity observed across the broader user base. This differentiation in activity patterns aligns with established understandings of ultradian rhythms in human behaviour, suggesting that the nature of user interaction with the forum—whether as consumers or producers of content—correlates with specific rhythmic cycles. Such insights into the temporal dynamics of user engagement not only underscore the influence of intrinsic biological rhythms on digital platform interaction but also highlight the potential for utilising these patterns in categorising user behaviours. Consequently, the identification of these distinct temporal patterns holds significant implications for law enforcement agencies in their efforts to combat unlawful activities associated with the use of digital platforms. By leveraging an understanding of these behavioural rhythms, strategies can be developed to more effectively identify and intervene in the activities of individuals engaged in illicit behaviour, thereby contributing to the broader efforts to mitigate the impact of such activities. This approach, grounded in the analysis of user activity patterns, offers a novel avenue for enhancing the efficacy of digital surveillance and enforcement mechanisms; other areas on online activity have explored the predictability of temporal patterns showing that the shorter the inter-event time, the less predictable are the individuals (or groups if we contextualise for our study) [29].

User and group differentiation arises not merely from temporal patterns of activity but also from the distinct content they produce and consume. Our analysis revealed significant variation in content preferences among users, with some engaging with a wide array of categories and others showing affinity for a limited selection. Utilising k-means clustering, we grouped users into eight clusters based on these preferences, noting that clusters 1, 2, and 3 predominantly engaged with a narrow range of content categories, whereas preferences in other clusters were markedly more diverse. The elbow method was employed to ascertain the optimal cluster count for the k-means algorithm. Analysis revealed a marked decrease in variance gain when *k* ≥ 8, prompting the selection of *k* = 8 as the ideal cluster number.

Understanding patterns of criminal behaviour is paramount, especially considering its contagious nature as highlighted by [30]. Recognising these patterns not only aids in identifying emergent trends but also enhances law enforcement capabilities to preempt and mitigate criminal activities. By analysing and understanding these trends, police forces can develop more effective strategies for early intervention, thus potentially curtailing harmful behaviours before they proliferate further. This approach is crucial for enhancing the effectiveness of prevention and enforcement measures against individuals involved in such activities. By identifying distinct patterns of behaviour, such as specific 24-hour, 12-hour, and 6-hour consumption cycles among users, as well as preferences for certain content categories, we offer law enforcement a nuanced understanding of offender dynamics. This knowledge enables the development of targeted surveillance techniques and intervention strategies, potentially increasing the effectiveness of early detection and prevention efforts. Moreover, our work underscores the importance of a multifaceted approach, combining data science and network analysis, to dissect the complex interactions within online communities. This holistic view not only aids in the more accurate profiling of individuals involved in CSEM but also supports the creation of tailored educational and intervention programs, aiming to disrupt these networks before they can expand further.

## Supporting information

S1 Appendix (PDF)
