# Supplementary material for: Unravelling the dynamics of child sexual exploitation material circulation on the Dark Web
Source: PLoS One. 2024 Jul 24;19(7):e0306516. doi: 10.1371/journal.pone.0306516 (PMC11268584; doi:10.1371/journal.pone.0306516)
Supplement: S1 Appendix — (PDF) [file pone.0306516.s001.pdf]

S1 Appendix.

Police Operation Setup. In 2014, the Cybercrime Unit of the Brazilian Federal Police at Rio Grande do Sul was authorised by a Brazilian Federal Court to infiltrate the Tor project to search for national individuals in activities related to child exploitation–it was the beginning of the so-called Operation DarkNet, one of the first investigations in the world to raid the Dark Web in search for illegal activities. Federal agents initiated surveillance on a forum within the Tor network, suspected of facilitating illegal activities. This meticulous investigation spanned approximately two years and unfolded in two distinct phases. Ultimately, it led to the identification of 182 individuals from a total of 10,407 users. This intensive inquiry resulted in the issuance of 156 search warrants, the arrest of 66 individuals, and the rescue of 6 children who had been confirmed as victims of abuse by some users of the forum. The execution of the warrants involved the coordinated efforts of over 500 Federal Agents and spanned 19 of Brazil's 27 states (including Brasília). In an international collaborative effort, information pertaining to non-Brazilian users was disseminated through Interpol to law enforcement agencies in Portugal, Italy, Colombia, Mexico, and Venezuela. Following this phase, the forum in question was shut down by court order, and all associated data were secured on police servers for detailed analysis. In a subsequent move, the court specifically authorised researchers to access the over 100 GB of collected data. This authorisation was granted to facilitate scientific and academic research aimed at developing innovative strategies for crime prevention and law enforcement.

An online forum, or message board, is a website where people can hold discussions and share information and media in the form of posted messages that are usually archived. In this kind of forum, topics (also called threads) are the lowest level places under which members (or users) hold their discussions or posts, which are user-submitted messages containing the timestamp of the submission as well as user's detail. Topics or threads are a collection of posts dedicated to a single conversation about a given subject. Two key roles are also important to distinguish: moderators, who are users with access to the content posted by all members to manage disputes, spams and organising the threads; and administrators, who can promote members to moderators and manage technical details necessary to running the message board. Infiltrated Federal Agents acted only passively on the illicit forum, not interfering on moderation, just gathering evidence on the crimes being committed. In this sense, all behaviour was emergent to the users themselves, without external influence. Precisely because of this, users split the forum into several categories (the top end of the tree-like directory structure of the forum) according to the type of discussion that was taking place and to the sort of media that was being shared.

COPINE. The COPINE (Combating Paedophile Information Networks in Europe) scale is a sophisticated framework developed to systematically classify child sexual abuse imagery, advancing from innocuous, non-sexualised depictions to those of severe abuse. Originating from the University of Cork, Ireland, this ten-tiered system plays a crucial role in both scholarly investigation and legal processes by providing a detailed criterion for evaluating the extent of exploitation captured in these images. By enabling a more precise characterisation of such material, the COPINE scale significantly aids in refining legal responses and therapeutic interventions. It embodies a concerted effort to address child sexual exploitation with a meticulous, standardised methodology, reflecting

a dedicated pursuit of protective measures for affected individuals through enhanced accuracy in identification and categorisation. By visually analysing the media shared on each of these categories, Federal Agents sorted the categories according to its predominant COPINE scale (see Table 1) aggregated over time at the day the forum was closed.

Table 1. The COPINE Scale. This classification is based on severity [1] and was proposed as part of the COPINE Project [2, 3]. We have adapted the scale and divided the grades into two groups we call non-sexual (from grade 1 to 6) and the Sexual group (starting at grade 7). This division is only used for the visualisation of the network in Fig 11; it does not affect the analyses.

Group	Grade	Descriptor	Description
Non-sexual	1	Indicative	Non-erotic and non-sexualised pictures showing children in their underwear, swimming costumes from either commercial sources or family albums. Pictures of children playing in normal settings, in which the context or organisation of pictures by the collector indicates inappropriateness.
	2	Nudist	Pictures of naked or semi-naked children in appropriate nudist settings, and from legitimate sources.
	3	Ero	Surreptitiously taken photographs of children in play areas or other safe environments showing either underwear or varying degrees of nakedness.
	4	Posing	Deliberately posed pictures of children fully clothed, partially clothed or naked (where the amount, context and organisation suggests sexual interest).
	5	Erotic posing	Deliberately posed pictures of fully, partially clothed or naked children in sexualised or provocative poses.
	6	Explicit erotic posing	Pictures emphasising genital areas, where the child is either naked, partially clothed or fully clothed.
Sexual	7	Explicit sexual activity	Pictures that depict touching, mutual and self-masturbation, oral sex and intercourse by a child, not involving an adult.
	8	Assault	Pictures of children being subject to a sexual assault, involving digital touching, involving an adult.
	9	Gross assault	Grossly obscene pictures of sexual assault, involving penetrative sex, masturbation or oral sex, involving an adult.
	10	Sadistic/Bestiality	Pictures showing a child being tied, bound, beaten, whipped or otherwise subject to something that implies pain or Pictures where an animal is involved in some form of sexual behaviour with a child.

Entropy Calculation. In our analysis, Shannon entropy serves as a tool to examine whether users–both consumers and producers of content–tend to focus their activities within a few forum categories or exhibit a broader diversity in their interests. This approach, however, does not account for the sequence in which categories are visited, focusing solely on the distribution of visits across categories. Future work could explore alternative entropy measures, such as Kolmogorov-Sinai or Lempel-Ziv entropies, to capture the dynamics of category visitation order, providing deeper insights into user behaviour patterns.

To assess the diversity of content categories within each cluster identified via k-means, we compute the entropy h for each cluster. For a given cluster c, encompassing n users where each user u_i accesses categories from set S with probabilities $p(s_j)$ for each

category s_j, the cluster's entropy is calculated as follows:

$$h(c) = -\sum_{j=1}^{|S|} p(s_j) \log_2 p(s_j).$$

Here, $p(s_j)$ denotes the likelihood of a user in cluster c engaging with category s_j, and $|S|$ represents the total category count. This probability is derived from the frequency of category visits by all users within the cluster. The entropy $h(c)$, therefore, reflects the diversity or uncertainty in content category preferences within the cluster, with higher entropy indicating a wider range of visited categories. This metric allows for an in-depth examination of content preference variations among different user groups, offering insights into user behaviour patterns on the forum. In Fig. 10 we can see how entropy informs our understanding of user engagement with diverse content categories.

References

1. Taylor M, Holland G, Quayle E. Typology of Paedophile Picture Collections. The Police Journal: Theory, Practice and Principles. 2001;74(2):97–107. doi:10.1177/0032258X0107400202.
2. Quayle E. The COPINE project. Irish Probation Journal. 2008;5(9):65–83.
3. Merdian HL, Thakker J, Wilson N, Boer D. Assessing the internal structure of the COPINE scale. Psychology, crime & law. 2013;19(1):21–34.
